# Editorial: Microbial Food Safety along the Dairy Chain

**DOI:** 10.3389/fmicb.2017.01612

**Published:** 2017-08-23

**Authors:** Edward M. Fox, Seamus Fanning, Aldo Corsetti, Kieran Jordan

**Affiliations:** ^1^CSIRO Agriculture and Food Werribee, VIC, Australia; ^2^UCD Centre for Food Safety; Science Centre South, University College Dublin Dublin, Ireland; ^3^Faculty of Bioscience and Technology for Food, Agriculture and Environment, University of Teramo Teramo, Italy; ^4^Teagasc Food Research Centre Cork, Ireland

**Keywords:** food safety, dairy

Milk is susceptible to contamination with pathogenic and spoilage organisms and, therefore, **Microbial food safety along the dairy chain** is an important topic, from public health and industry perspectives. The dairy chain is an integral part of global food supply, with dairy food products a staple component of recommended healthy diets. The dairy food chain from production through to the consumer is complex, with various opportunities for microbial contamination of ingredients or food products, and as such interventions are key to preventing or controlling such contamination. Dairy foods often include a microbial control step in their production such as pasteurization, but in some cases may not, as with raw milk products. Microbial contamination may lead to a deterioration in food quality due to spoilage organisms, or may become a health risk to consumers should the contaminant be a pathogenic microorganism. As such food safety and food production are intrinsically linked.

This e-Book brings together a series of articles related to the microbiological integrity of the dairy food chain, with regard to pathogenic and spoilage organisms, genomic and other analyses of these contaminants, and alternative methods for their study.

The first two papers concern sporeforming bacteria, which are a concern both from safety and processing perspectives. The paper McHugh et al. the specifications for spores in milk powders can be low and so traditional detection methods used in industry to enumerate them have limitations in terms of time, efficiency, accuracy, and sensitivity. The review provides an insight into recent advances in methodology for detection of spores, highlighting the advantages and limitations with respect to the application of such methodologies for dairy food. Optimisation and application of these methods can ensure safety and quality standards.

The paper Doll et al. is more specific. It addresses the issue of the spoilage of extended shelf life milk and concluded that the shelf-life was not influenced to a large extent by raw-milk-associated factors, but by recontamination with spores, particularly from the *B. cereus* complex. To enhance milk quality throughout the entire shelf life, improved plant sanitation and disinfection that target the elimination of spores are necessary.

Machado et al. is a review of the growth potential of psychrotrophic bacteria, the heat resistant enzymes they produce and the consequences for dairy products with a long shelf life. Due to their ability to produce extracellular heat resistant enzymes such as peptidases and lipases, psychrotrophic bacteria, such as species of *Pseudomonas*, can contribute to spoilage of ultra-high temperature (UHT) treated and sterilized milk and other dairy products with a long shelf life. This problem is of increasing importance because of the large worldwide trade in fluid milk and milk powder.

For many years, coliforms have been used as indicators of contamination of dairy products, and continue to be used for this purpose. Martin et al. highlights that recent discoveries regarding this diverse group of bacteria indicates that only some are of fecal in origin, while the majority are environmental contaminants, raising questions regarding the validity of coliforms as indicators of unhygienic conditions for dairy products. The role that coliforms play in raw and finished dairy products, their sources and the future of this diverse group as indicator organisms in dairy products is discussed.

The next six papers deal specifically with foodborne pathogens; three with *S. aureus*, and one with each of the pathogens *Salmonella*, pathogenic *E. coli* and *L. monocytogenes*.

The paper Merz et al. determined that *S. aureus* isolates from sheep and goats were similar, but were different from bovine isolates. Sixty-seven percent of the *S. aureus* strains detected exhibited at least one enterotoxin gene, indicating that many caprine, or ovine raw milk products may be contaminated with low levels of enterotoxigenic *S. aureus*, stressing the importance of strict maintenance of the cold chain.

The paper Kümmel et al. showed that dairy cattle represent an important, yet underreported, entrance point of *S. aureus* into the dairy chain. It was shown that certain *S. aureus* subtypes were present in primary production as well as in the cheese processing at the dairy plant and although a considerable diversity of *S. aureus* subtypes was observed, only certain *S. aureus* subtypes were able to enter and persist in the cheese manufacturing at the dairy plant, and could be isolated from cheese up to day 14 of ripening.

The paper Yu et al. concerns the control of *S. aureus* infection in cows. They demonstrated that the *in vivo* bacterial killing activity elevated when dosage increased or when dosing intervals were shortened, and recommended a regimen of three infusions of 75 mg per quarter every 12 h to achieve a 76.7% cure rate in clinical treatment of bovine mastitis caused by *S. aureus* infection.

The paper Gunn et al. used molecular characterization to relate two historical outbreaks associated with *Salmonella* serovars Anatum and Ealing. Pulsed-field gel electrophoresis (PFGE) revealed the clonal nature of the two outbreaks and whole genome sequencing (WGS) of representative isolates, one from each serovar, focused on the *Salmonella* pathogenicity islands. The results suggested a high level of genetic diversity that may have contributed to survival and virulence of isolates from these outbreaks.

Shiga toxin-producing *E. coli* (STEC) are significant pathogens in the dairy chain. The paper Murphy et al. examined the issue of super-shedding (shedding of >10,000 CFU/g of feces) of *E. coli* O157 and O26 by lactating cows and its impact (if any) on raw milk. The results showed that of the 529 samples taken from 40 animals, 4 animals were deemed as super-shedders, one shedding *E. coli* O157 and 3 shedding *E. coli* O26. No STEC O157 or O26 were recovered from any of the raw milk, milk filter, or water samples. The results show that vigilance is required with regard to super-shedding of non-O157 STEC.

Another important pathogen in the dairy chain is *L. monocytogenes*. The paper Casey et al. contributes to understanding the pathogenicity of *L. monocytogenes*. Two serotype 1/2b strains of *L. monocytogenes* with differing infection abilities were subjected to comparative genomic analysis. The results showed the importance of accessory genes (genes that are not part of the conserved core genome) in *L. monocytogenes* pathogenesis and suggested that the emergence of an apparently non-pathogenic isolate of *L. monocytogenes* may result from a cumulative loss of functionality rather than by a single isolated genetic event.

Biofilm formation is an important factor in the community life of bacteria in the environment, providing protection against inactivation processes for pathogenic bacteria. The paper Miljkovic et al. demonstrated that deletion of the collagen binding repeats II, III, and IV, necessary for auto-aggregation, resulted in a loss of the strong auto-aggregation, collagen and fibronectin binding abilities whereas the biofilm forming capability was increased.

In the final paper, Anvarian et al. a combination of flow cytometry (FCM) and 16S rDNA sequencing was used to investigate the microbiome in a powdered infant formula (PIF) production facility. The greatest diversity in the microbiome was observed in the low care area. The genera present in low, medium and high care were mostly associated with soil, water, and humans, respectively. The integration of FCM and metagenomic data provided information on the density of different species in the facility.

## Author contributions

All authors listed have made a substantial, direct and intellectual contribution to the work, and approved it for publication.

### Conflict of interest statement

The authors declare that the research was conducted in the absence of any commercial or financial relationships that could be construed as a potential conflict of interest.

